# The Epistatic Landscape of Antibiotic Resistance of Different Clades of *Mycobacterium tuberculosis*

**DOI:** 10.3390/antibiotics10070857

**Published:** 2021-07-15

**Authors:** Dillon Muzondiwa, Hleliwe Hlanze, Oleg N. Reva

**Affiliations:** Centre for Bioinformatics and Computational Biology, Department of Biochemistry, Genetics and Microbiology, University of Pretoria, Pretoria 0002, South Africa; dillonmuzondiwa@gmail.com (D.M.); ntasenahle@gmail.com (H.H.)

**Keywords:** *Mycobacterium tuberculosis*, antibiotic resistance, epistasis, drug-resistant mutation, evolution

## Abstract

Drug resistance (DR) remains a global challenge in tuberculosis (TB) control. In order to develop molecular-based diagnostic methods to replace the traditional culture-based diagnostics, there is a need for a thorough understanding of the processes that govern TB drug resistance. The use of whole-genome sequencing coupled with statistical and computational methods has shown great potential in unraveling the complexity of the evolution of DR-TB. In this study, we took an innovative approach that sought to determine nonrandom associations between polymorphic sites in *Mycobacterium tuberculosis* (Mtb) genomes. Attributable risk statistics were applied to identify the epistatic determinants of DR in different clades of Mtb and the possible evolutionary pathways of DR development. It was found that different lineages of Mtb exploited different evolutionary trajectories towards multidrug resistance and compensatory evolution to reduce the DR-associated fitness cost. Epistasis of DR acquisition is a new area of research that will aid in the better understanding of evolutionary biological processes and allow predicting upcoming multidrug-resistant pathogens before a new outbreak strikes humanity.

## 1. Introduction

Despite being a disease of antiquity, TB caused by *Mycobacterium tuberculosis* (Mtb) remains one of the leading killers globally, with approximately 10 million new cases annually and about 2 billion people infected globally [1]. While a global decrease in TB incidence is reported, drug-resistant TB (DR-TB) has been identified as a cause for concern in global efforts to eradicate TB [2]. According to a report by the World Health Organization (WHO) in 2017, approximately 4.1% of new TB cases and 19% of previously treated cases were classified as multidrug-resistant TB (MDR-TB). MDR-TB can be classified as resistance to first-line drugs isoniazid and rifampicin, and ultimately, extensive drug resistance (XDR) is resistance to first-line drugs and at least one second-line drug, i.e., amikacin, kanamycin, capreomycin or one of the fluoroquinolones [3,4]. Treatment for patients with MDR and XDR is often accompanied by prolonged and costly antibiotic courses and poor outcomes that result in high rates of mortality and treatment failure [5,6]. Countries that have a high burden of total cases of TB including DR-TB are often resource-limited. Consequently, the inadequate administration of treatment risks the amplification of resistance to further drugs and may prolong opportunities for infection transmission [7]. Timely diagnosis and correct treatment are of the utmost importance in the global fight against TB. DR-TB can be acquired during treatment through the acquisition of chromosomal mutations in drug target genes (secondary resistance) or can be transmitted from one individual to another (primary resistance) [6,8]. The development of acquired resistance is common as a result of single-nucleotide polymorphisms (SNPs), insertions and deletions [5,7]. Apart from chromosomal mutations, the intrinsic resistance due to the bacterium’s lipid-rich cell wall and efflux pumps has limited the number of drugs that can treat TB [5,9]. Rapid molecular diagnostic assays have been developed to tackle the challenge of early diagnosis and treatment. However, the technology behind these trials is often limited to a small number of loci they can interrogate, and thus, the number of drugs that can be tested usually is restricted to first-line drugs [10,11]. The genetic basis of drug resistance is still poorly understood, with recent research suggesting that it is more complex than previously thought [12]. Whole-genome sequencing (WGS) technology has proven to be an effective tool in shedding more light on our understanding of TB [13]. Unlike in most bacterial pathogens, horizontal gene transfer does not play a role in the acquisition of DR in Mtb. For this reason, research has mostly focused on identifying multivariate associations between SNPs in the Mtb genome and the resistant phenotype. The application of mathematical methods in tandem with WGS and other related TB data has also proved to be vital in exploring the associations between TB variants [5,14,15,16].

Focusing exclusively on DR-associated mutations uncovers the whole complexity of the antibiotic resistance evolution in TB. Patterns of distribution of DR mutations in TB genomes appeared to be lineage specific due to epistasis limitations [3,17,18]. When a DR mutation is acquired, it is expected that this would impose a fitness cost. However, a number of studies have shown that MDR strains have low or no fitness cost, yet they show a high level of resistance [10,18,19,20]. Several DR mutations in genes *katG*, *gyrA*, *rpoB* and *rpsL* render resistance to different antibiotics and are frequently observed in successful DR clinical isolates [18]. On the other hand, a household-based case study conducted by Salvatore et al. [21] demonstrated that isolates carrying both *katG* S315T and *rpsL* R43K mutations were less likely to be found in households with multiple cases, suggesting decreased transmission even though both mutations conferred high-level resistance. Several studies have identified putative compensatory mutations in *rpoA*, *rpoC* and *rpoB* genes that ameliorate the fitness cost of rifampicin resistance [22,23]. Newer work conducted by Zimic et al. [24] identified 35 additional putatively novel compensatory mutations in *rpoC* genes from isolates that were rifampicin resistant in Peru. Another study conducted by Coll et al. [5] identified a *pnaB2* mutation that may compensate for pyrazinamide resistance conferred by *pncA* and mutations in the *thyX–hsdS.1* promoter that compensates for para-aminosalicylic acid resistance conferred by mutations in *thyA*. The stated examples suggest that these strains may have undergone compensatory evolution modulating the fitness of the strains, which either precedes or follows the drug resistance acquisition by these strains [3,25,26].

Another class of steppingstone mutations that emerge before antibiotic resistance has been acquired has been reported in a study by Cohen et al. [11]. These authors found that mutations in *ubiA* emerge before ethambutol resistance conferred by mutations in *embB*. Safi et al. [27] showed *in vitro* a multistep selection of *ubiA*, *aftA*, *embB* and *embC* mutations required to achieve the highest level of resistance to ethambutol. Recently, a study conducted by Shea et al. [28] identified low-level resistance conferring mutations in the *rpoB* gene. Associations between the genetic background and DR have been investigated in several studies [3,25,27,29]. For example, Li et al. [30] found that substitutions at *katG*315 and *rpoB*450 loci conferring rifampicin resistance were more frequent in more transmissible strains of the “modern” Beijing sublineages compared to the “ancient” Beijing strains. Additionally, Fenner et al. [31] found that mutations in *katG* and *inhA* conferred different levels of INH resistance depending on the lineage belonging to the mutant TB strains. In Lineage-2 mutants, substitutions in *katG*315 were more prevalent and conferred high-level resistance to isoniazid, whereas in the Lineage-1 strains, more prevalent were substitutions in *inhA* conferring low-level resistance to isoniazid. Several studies have highlighted the effectiveness of WGS in predicting resistance to mostly first-line anti-TB drugs. However, studies that attempt to predict resistance to second-line drugs have been limiting [32,33]. Before WGS-based diagnosis can be implemented in clinical laboratories, there is a need to update the current catalog of determinants of resistance to cater to second-line drugs. This can be achieved by resolving some of the elusive genotype and phenotype correlations through the application of statistical and computational methods, especially for drugs such as pyrazinamide, where phenotypic drug susceptibility testing is challenging [11,32]. Understanding the stepwise molecular processes of the accumulation of initial prerequisite mutations acting as a gateway for the emergence of MDR is another gap in our knowledge [34]. Knowing the ways of escaping the fitness cost of DR mutations by MDR-TB will uncover molecular targets for new antibiotics and allow predicting Mtb clinical isolates with a potential to develop MDR in the near future [35].

In this study, we performed a systemic analysis of compensatory mutations associated with several TB lineages, which often cause MDR-TB infections.

## 2. Results

This study includes an analysis of 2501 Mtb isolates available from the GMTV database, which underwent culture-based drug susceptibility testing and lineage classification [36]. Pairs of polymorphic sites within selected Mtb genomes showing a nonrandom distribution were analyzed using Levin’s attributable risk statistic [37], which predicts the likelihood of a subordinate substitution of the allelic state *B* to the state *b* if a primary mutation at another locus from the allelic state *A* to the state *a* already took place. This likelihood was denoted as *B→b_|a_*. An opposite relation when the mutation at the locus *B* induces the secondary mutation at the locus *A*, denoted as *A→a_|b_*, was also considered. If the likelihoods *A→a_|b_* and *B→b_|a_* are comparable, it may indicate a genetic drift of two independent mutations. If *B→b_|a_* is significantly greater than *A→a_|b_*, it indicates that the mutation *A→a* was a prerequisite for the secondary mutation *B→b*. It should be noted that in this study, the allelic states A and B were the same as in the reference genome H37Rv (NC_000962.3), and the allelic states *a* and *b* were other possible alternatives identified in the sequenced genomes. While many associations between polymorphic sites of Mtb genomes were found, in this study, we paid attention only to the epistatic links associated with known DR mutations [38]. Particularly, we considered epistatic links to mutations rendering resistance against ethambutol (EMB), ethionamide (ETH), isoniazid (INH), fluoroquinolones (FLQ), pyrazinamide (PZA), rifampicin (RIF) and streptomycin (SM). Assuming that the DR acquisition in different lineages of Mtb exploited different evolutionary pathways [3], the selected Mtb genomes were grouped by their lineage belonging before the analysis. The results of the computational reconstruction of DR-related epistatic networks of several Mtb clades are presented below. In this study, we focused only on sense mutations rendering amino acid replacements in the coding proteins. The mutations were referred to in the format *gene_name*:#, where # is the number of the polymorphic codon.

### 2.1. Lineage 1: East African and Indian Clade

**Figure 1:**
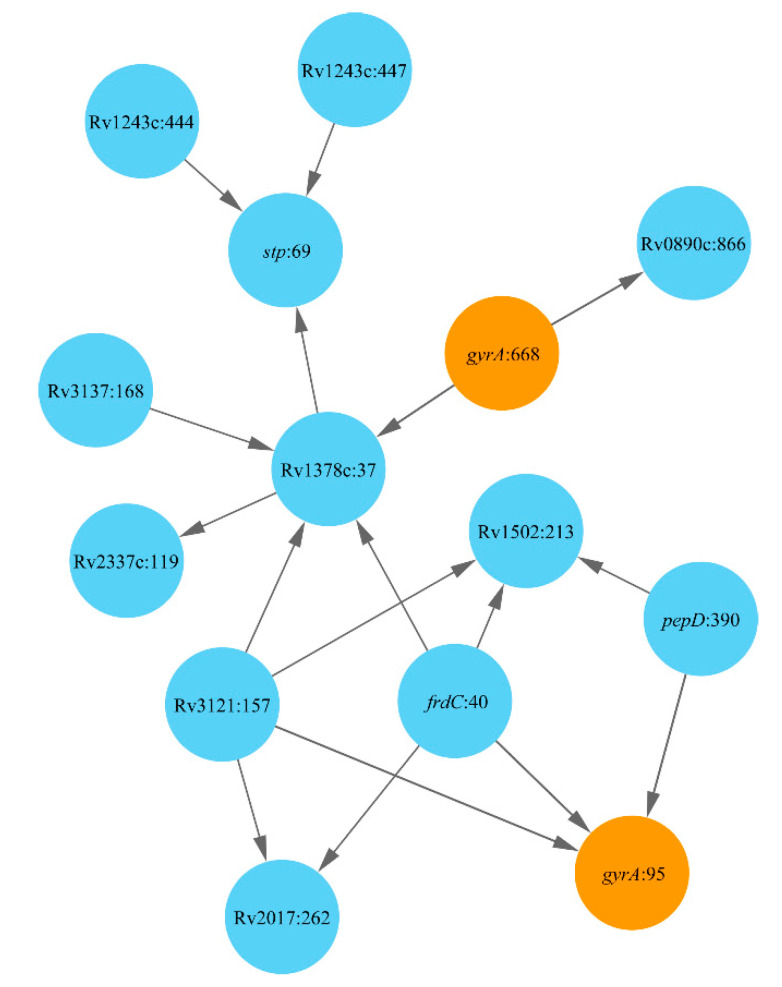
Network of DR-associated mutations in Lineage 1.2. Polymorphic loci are shown as nodes titled by the respective gene name and the polymorphic codon number following the colon. Mutations known to cause DR are depicted by color. Arrows show relations between prerequisite and subordinate mutations.

Strains of Lineage 1 are generally known to be drug susceptible [39], except for Sublineage 1.2 considered in this section. Paths connecting the DR mutations in Mtb strains of this lineage are shown in Figure 1. Two polymorphisms at codons 95 and 668 were found in the known drug target *gyrA*. Both these mutations are known to be associated with resistance to FLQ [40]. Mutation in the *frdC*:40 may be a prerequisite to the acquisition of the antibiotic resistance mutation *gyrA*:95. The gene *frdC* is known to affect quinol binding [41], although there is a need to investigate its possible role in Mtb drug resistance. Mutation *pepD*:390 also preceded the acquisition of *gyrA*:95 mutation in this Mtb clade. The gene *pepD* is a stress response protein whose loss of function subsequently affects the expression of other stress response determinants in Mtb [42]. Another FLQ resistance mutation *gyrA*:668 has appeared independently in the population and led to several compensatory mutations in transcriptional regulator Rv0890c, conserved hypothetical protein Rv13178c and several other protein-coding genes, including *stp*, an efflux pump [43], which may compensate for the loss of fitness that might result from the mutations in *gyrA*.

### 2.2. Lineage 2: Beijing Clade

**Figure 2:**
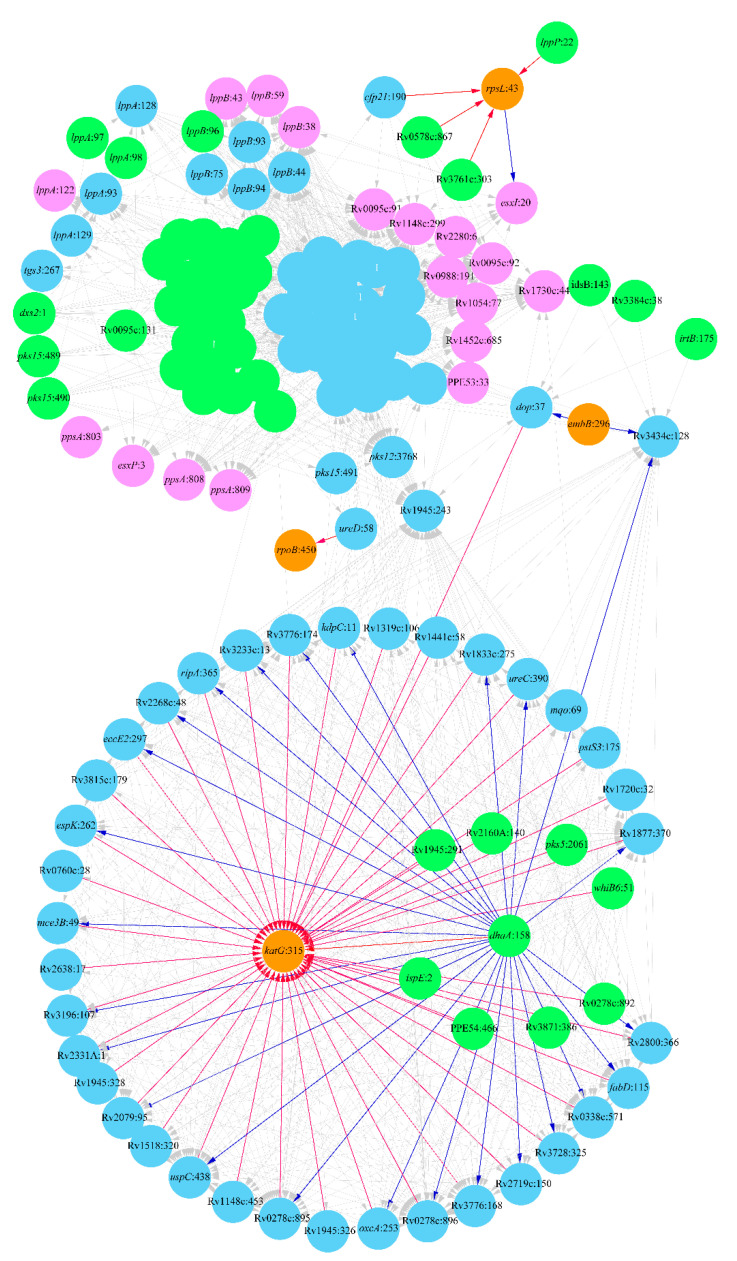
Network of DR-associated mutations in the clade Beijing (Lineage 2). Polymorphic loci are shown as nodes titled by the respective gene name and the polymorphic codon number following the colon. Mutations known to cause DR are depicted by an orange color. Nodes linked exclusively by outgoing edges (prerequisite mutations) are shown in green, and those linked exclusively by incoming edges (final and compensatory mutations) are shown in pink, while intermediate mutations are shown in blue. Arrows show the relations between prerequisite and subordinate mutations, which in the case of direct association with the known DR mutations are highlighted by red and blue colors, respectively. The mutations linked to more than 20 subordinate mutations and having no prerequisite mutations were supposed as starting points of the DR evolution. They are depicted by a red stroke outline.

The Beijing clade has been associated with hypervirulence and propensity to develop MDR-TB. The analysis showed a complex evolutionary network of mutations co-occurring with antibiotic resistance mutations (Figure 2). It seems that one of the central events that started the evolutionary processes towards MDR development was the mutation *dhaA*:158 that induced multiple subordinate mutations in various genes. The gene *dhaA* encodes a haloalkane dehalogenase. This gene is upregulated in bacteria living inside macrophages [44]. Other five initial mutations of this pull, which induced more than 20 subordinate mutations, were in 4-diphosphocytidyl-2-C-methyl-D-erythritol kinase IspE, ESX-1 type VII secretion system protein Rv3871, transcriptional regulatory protein WhiB6, polyketide synthase Pks5 and uncharacterized protein Rv2160A (Figure 2). All these mutations created the epistatic background for the acquisition of DR mutations *katG*:315, *embB*:296, *rpoB*:450 and *rpsL*:43.

Mutations in the genes of the urease locus, *ureD*:58 and *ureC*:390, were prerequisites for the antibiotic resistance mutations *rpoB*:450 and *katG*:315 causing resistance to FLQ and INH, respectively. The urease locus of Mtb is an important virulence factor to resist acidity and maintain intracellular pH in macrophages [45]. More than 43 mutations in various genes were prerequisites for the *katG*:315 mutation, including the potassium-transporting subunit *kdpC*, which is important for surviving antibiotic treatment and persister Mtb formation [46]; malonyl CoA-acyl carrier protein transacylase *fabD* involved in fatty acid and mycolic acid synthesis [47]; oxalyl-CoA decarboxylase *oxcA*, whose epistatic interaction with *katG* was predicted in another study [48]; virulence-associated type VII secretion system protein *eccE2*; peptidoglycan hydrolase *ripA* essential for drug resistance and persistence of Mtb [49]; and other genes, whose involvement in antibiotic resistance should be clarified in further studies.

The *dop* deamidase encodes an enzyme of the pupylation pathway that plays an important role during the intracellular persistence of Mtb and supports the resistance of this pathogen towards oxidative and nitrosative stress encountered inside the host macrophages [50]. This study showed that the *dop*:37 mutation has an epistatic link to *katG*:315 and *embB*:296 INH and EMB resistance mutations. Another compensatory mutation following the *embB*:296 EMB resistance mutation is Rv3424c:128 within an uncharacterized conserved transmembrane protein.

The SM resistance mutation *rpsL*:43 shows epistatic relations in Beijing clade MRD isolates with mutations in cutinase *cfp21*:190, acyl-CoA dehydrogenase *fadE36*:303, lipoprotein *lppP*:22 and PE-PGRS family protein Rv0578c, which were prerequisites of this mutation. The polymorphic site in ESAT-6 like protein *esxI*:20 serves as a compensatory mutation.

Many other genes create the epistasis of MDR strains of the Beijing clade (Figure 2). Many clade-specific mutations associated with DR were found in genes *lppA* and *lppB* encoding lipoproteins, which play an important role in the synthesis of cell wall fatty acids of bacteria [51]. A study by Torrey et al. [52] identified a non-synonymous mutation in *lppA* in a high-persister Mtb mutant. Three compensatory mutations were found in phenolpthiocerol synthesis type-I polyketide synthase *ppsA* in codons 803, 808 and 809 and in several other polyketide synthases: *pks12* and *pks15*. The *ppsA-E* genes are responsible for the synthesis of the virulence factor pthiocerol dimycocerosate, and they are significantly upregulated in MDR Beijing Mtb isolates [53]. All these mutations created epistatic links to a pull of many other mutations in various genes, which only indirectly may be related to the DR phenotype. In Figure 2, these mutations are shown as untitled nodes, except for the central mutation in the uncharacterized hypothetical protein Rv0095c, which is the starting point of the mutations of this pull.

### 2.3. Lineage 4: Haarlem and Ural Clades

Lineage 4, known also as European-American lineage, is the most widely distributed modern clade of *M. tuberculosis* that causes acute and antibiotic-resistant tuberculosis nowadays on all the continents due to human migrations [54]. This lineage is subdivided into several sublineages associated with different geographical areas. In this study, the evolution of antibiotic resistance was analyzed in two sister sublineages of Lineage 4 [55]: North American clade Haarlem and clade Ural, which is common in the central part of Russia.

#### 2.3.1. Clade Haarlem

**Figure 3:**
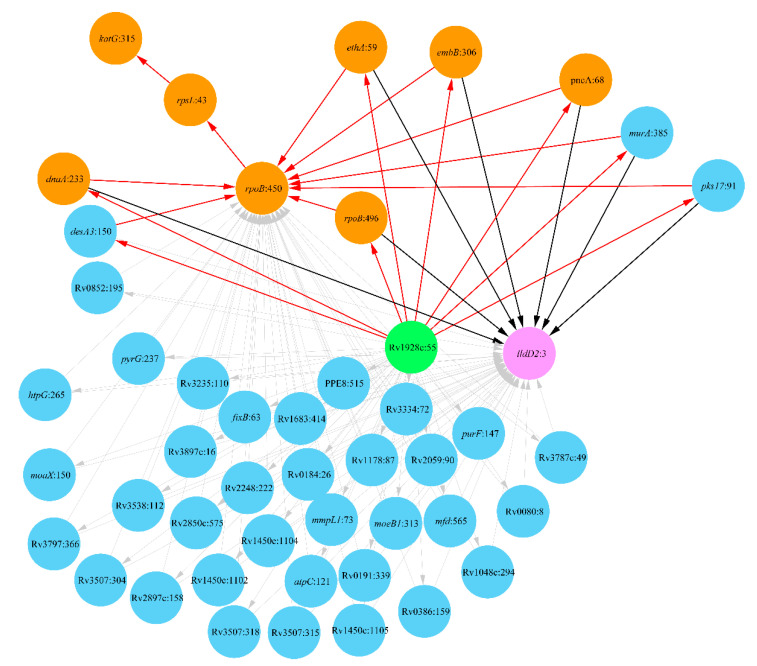
Network of DR-associated mutations in the clade Haarlem (Lineage 4.3). Polymorphic loci are shown as nodes titled by the respective gene name and the polymorphic codon number following the colon. Mutations known to cause DR are depicted by an orange color. Nodes linked exclusively by outgoing edges (prerequisite mutations) are shown in green, and those linked exclusively by incoming edges (final and compensatory mutations) are shown in pink, while intermediate mutations are shown in blue. Arrows show relations between prerequisite and subordinate mutations. Major steps of DR evolution in the Haarlem clade are shown as sick red arrows, and the directions to the major compensatory mutation *lldD2*:3 are shown as sick black arrows.

Analysis of the distribution of DR mutations among Haarlem strains showed several stages of the acquisition of multiple antibiotic resistance by these strains as shown in Figure 3.

The evolution of antibiotic resistance started from a mutation in codon 55 in a putative dehydrogenase/reductase Rv1928c whose involvement in INH resistance was hypothesized in a recent paper [56]. This mutation made it possible for several other DR mutations to occur independently from each other: *rpoB*:496 for RIF resistance [57], *dnaA*:233 for INH resistance [58], *ethA*:59 for ethionamide resistance [59], *embB*:306 for EMB resistance [60] and *pncA*:68 for pyrazinamide [61]. The mutation Rv1928c:55 was a prerequisite for many other subordinate mutations, particularly in *murA* encoding a key enzyme of peptidoglycan biosynthesis, polyketide synthase *pks17* and *desA3* predicted as a potential DR gene [56]. The evolution of antibiotic resistance in the Haarlem clade followed several continuous steps of acquisitions of further known DR mutations: *rpoB*:450, *rpsL*:43 and *katG*:315. These mutations enforced the resistance against RIF and rendered the strains with an additional resistance against SM and INH. Genetic polymorphism in gene *lldD2* encoding lactate dehydrogenase ended up the evolution of MDR Haarlem strains possibly by providing a compensatory modification. The importance of this gene in the survival of Mtb macrophages is known, and the polymorphism *lldD2*:3 is typical for Lineage 4 [54].

This antibiotic resistance evolutionary pathway was accompanied by several other mutations in various genes, which finalized the specific epistatic landscape of the MDR Haarlem strains. Mutated genes included several efflux pumps, *mmpl* and *fadD*, which may also play a compensatory role in the development of MDR-TB. Many other epistatic mutations in protein-coding genes are shown in Figure 3.

#### 2.3.2. Clade Ural

**Figure 4:**
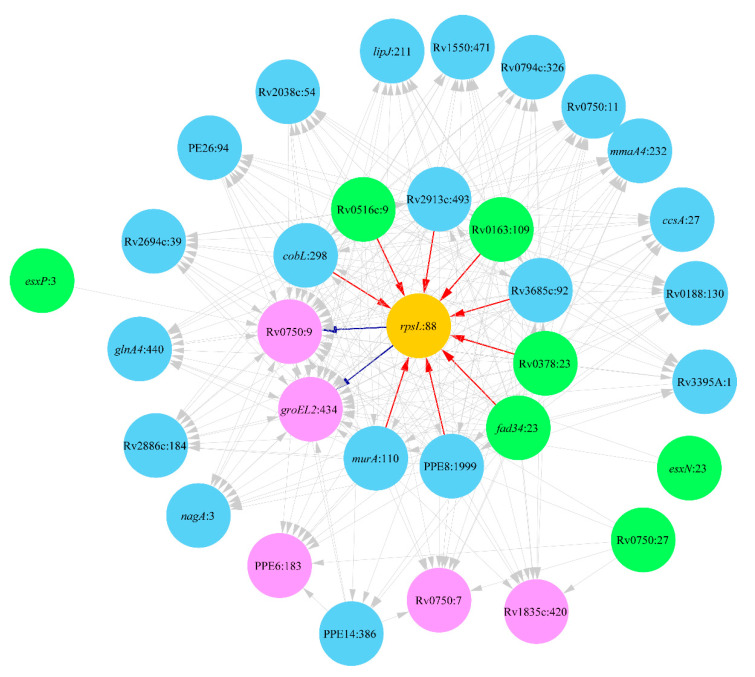
Network of DR-associated mutations in the clade Ural (Lineage 4.2). Polymorphic loci are shown as nodes titled by the respective gene name and the polymorphic codon number following the colon. DR mutation *rpsL*:88 is depicted by an orange color. Nodes linked exclusively by outgoing edges (prerequisite mutations) are shown in green and those linked exclusively by incoming edges (final and compensatory mutations) are shown in pink, while intermediate mutations are shown in blue. Arrows show relations between prerequisite and subordinate mutations, which in the case of direct association with the DR mutation are highlighted by red and blue colors, respectively.

Analysis of Mtb strains of the clade Ural revealed several epistatic links towards SM resistance evolution associated with the mutation *rpsL*:88 characteristic for this clade [62]. The predicted network is shown in Figure 4.

Mutations in nine genes were direct prerequisites of the acquisition of the DR mutation *rpsL*:88. They include mutation Rv0516c:9 in the gene associated with the responses to osmotic stress, whose disruption enhances DR [63]. The mutation in *murA* was a prerequisite for the DR development in Haarlem strains. The genetic polymorphism in this gene is also important for DR evolution in the clade Ural; however, it occurs at an alternative codon 110. Other involved genes were cobalamin synthesis enzyme *cobL*, cytochrome *cyp137* (Rv3685c), fatty-acid-CoA ligase *fadD34* and several uncharacterized proteins. The DR mutation *rpsL*:88 induced at least two compensatory mutations in chaperonin *gloEL2* and uncharacterized conserved protein Rv0750. Chaperonin *gloEL2* was implicated in the development of resistance to aminoglycosides [64,65]. Aminoglycosides, such as streptomycin, are known to exert their effect by causing a translational misreading [66]. It may be assumed that the avulsion of the resistance to aminoglycosides needs some compensatory mutations in this chaperonin. The mutations, which were prerequisites for the DR mutation *rpsL*:88, in turn, induced an accumulation of several other compensatory mutations shown in Figure 4. Among them, there are noteworthy mutations in methoxy mycolic acid synthase *mmaA4*, cytochrome C-type biogenesis protein *ccsA*, peroxidase *lipJ*, glutamine synthetase *glnA4*, N-acetylglucosamine-6-phosphate deacetylase *nagA* and ESAT-6 like proteins *esxN* and *esxP*.

## 3. Discussion

Several factors, which include fitness cost, compensatory mechanisms and phylogenetic background, have been shown to play important roles in the evolution of drug resistance in Mtb. However, current scientific tools cannot directly measure some of these factors experimentally, hence we have to rely on mathematical modeling to elucidate the relationships between these factors and how they influence the DR phenotype in Mtb. These approaches have also been successful in challenging some of the traditional dogmas that have governed TB research for years. For example, earlier studies have always assumed that all DR Mtb strains possess a fitness disadvantage when compared to their susceptible counterparts. Contrary to that view, mathematical modeling and experimental studies have proved that bacterial fitness is a heterogeneous entity [67]. In this study, we coupled statistical modeling with WGS and drug susceptibility (DST) data to model functional associations between polymorphisms that show epistatic links to the DR phenotype. Epistatic mutations were found in many genes involved in stress response, efflux pumping, lipid metabolism, cell wall synthesis and transportation, DNA repair, etc.

From this study, it can be hypothesized that efflux pumps facilitate the tolerance to isoniazid in Mtb strains. The loss of *katG* function leads to an impairment in the system that protects Mtb from reactive oxygen species (ROS). This leaves Mtb vulnerable to further damage from ROS and other antibiotics. DNA damage in bacteria is known to induce SOS response in an attempt to repair the damage. However, this system leads to elevated mutation rates, as it induces DNA polymerase II, IV and VI, which are highly associated with errors. The increased mutation rate in turn results in the accumulation of further mutations in the MTB genome, as it evolved into high levels of MDR-TB/XDR-TB. It is also important to note that the use of rifampicin directly induces the SOS mechanism and therefore promotes Mtb mutagenesis. Aminoglycosides such as kanamycin and streptomycin also activate the system by inducing translational stress, which might explain why the *rpsL* gene was a central point in the evolution of DR in Mtb strains. A study by Javid et al. [68] implicated this gene as a steppingstone to DR development due to destabilizing the flow of genetic information transformation (DNA-RNA-protein), resulting in an increased genetic and proteomic polymorphism.

Lipid metabolism genes also seem to play a leading role in the DR evolution in all Mtb clades. Mutations in these genes modify their enzymatic activities and therefore compensate for the loss of fitness caused by the acquisition of DR mutations. Drug tolerance mechanisms seem to provide the initial stepping platform that ushers the beginning of the path to DR evolution.

In this study, Levin’s attributable risk statistic [37] was used to identify pairs of linked genetic polymorphisms and the direction of evolution from one to another mutation. The analysis revealed networks of mutations associated with the evolution of DR against several antibiotics in different Mtb clades. These results are not comprehensive. Many more mutations associated with DR are known from the literature, which were not analyzed in this study due to some limitations of the used approach. Attributable risks are predicted based on a given training set of sequenced genomes provided with some metadata on strain-specific antibiotic resistance patterns. While there are many completely sequenced Mtb genomes in public databases, only a few of them are adequately characterized regarding their phenotypes. The best statistical reliability of calculations may be achieved when the training dataset is sufficiently large and polymorphic sites of interest are represented by alternative alleles with half-to-half frequencies. Obviously, it is impossible to create such a dataset that will work for all DR-related polymorphisms. Some of them will be missed, while others will be overrepresented due to biases in public databases towards sequences of antibiotic-resistant isolates. Another problem with these statistics is the possible admixture of the training datasets. Most often around the world tuberculosis is caused by Mtb strains belonging to the clade LAM that is a sublineage of Lineage 4. The worldwide expansion of this clade historically originated from Europe was explained by continuing human migrations [54]. While these bacteria are eager to develop DR, the network generated by the attributable risk statistics analysis was rather complex, with links to the known DR mutations showing rather weak statistical reliability. Eventually, we decided to exclude these results and leave them for further detailed analysis. A possible reason for this failure is that the DR development by the strains of this clade may follow different evolutionary pathways in Europe, Latin America and Africa due to differences in antibiotic treatment regimens, different adherence of patients to the treatment and different human genetic backgrounds. The attributable risk statistics returns the best results when the selected isolates are transmissibly linked to each other. Overcomplexity of the DR epistasis network estimated for the clade Beijing (Figure 2) may also be associated with the fact of the global expansion of these pathogens around the world, which led to a large variety of DR evolutionary pathways.

The current advance in WGS technology shows a drop in costs and an improvement in the quality of data generated. This means that it is now possible to simultaneously sequence hundreds or even thousands of isolates cheaply. Long-read sequencing technology also promises to address some of the challenges that have traditionally been associated with characterizing the highly repetitive regions of the Mtb genome. These developments will help to guide our future plans that seek to address the limitations associated with the attributable risk statistic applications. Studies on epistatic networks relevant to DR and virulence evolution will allow predicting outbreaks of DR infections in advance at the time of formation of an epistatic background of antibiotic resistance. This idea was realized in our recent software tool, Resistance Sniffer, which uses NGS data to predict Mtb isolates, which potentially may develop DR in the course of antibiotic therapy [35].

## 4. Materials and Methods

### 4.1. Data Sourcing

Data for this project were obtained from the GMTV database [36], which provides a comprehensive catalog of variant call format (VCF) files and metadata information on phenotypic drug susceptibility testing (pDST) and lineage classification for each sample. The collated data of 2501 Mtb strains were then used to calculate the mutation frequencies for the selected Mtb clades.

### 4.2. Functional Associations between Mtb Mutations

Borrowing from an earlier paper by van Nierkerk et al. [3], Levin’s attributable risk statistic (*R_a_*-statistic) was employed for this study [37]. The *R_a_*-statistic was used to identify directional epistatic relations and separate them from genetic drift associations between mutations in the Mtb genomes by using Equation (1) for the statistical estimation of attributable risks and Equation (2) for the Fleiss’ standard error estimation. Allele combination frequencies are denoted as *P_AB_*, *P_Ab_*, *P_aB_* and *P_ab_*, while *N* represents the total number of the analyzed Mtb strains.

(1) $$R_{A\to a|b}=\frac{P_{AB}P_{ab}-P_{aB}P_{Ab}}{\left(P_{AB}+P_{aB}\right)\left(P_{aB}+P_{ab}\right)}$$

(2) $$StdErr=\sqrt{\frac{P_{Ab}+R_{aA\to a|b}\left(P_{AB}+P_{ab}\right)}{N\times P_{aB}}}$$

The likelihood and the standard error of an opposite relation of the mutation *B→b* from the mutation *A→a* were calculated by using Equations (3) and (4), respectively.

(3) $$R_{B\to b|a}=\frac{P_{AB}P_{ab}-P_{aB}P_{Ab}}{\left(P_{AB}+P_{Ab})(P_{Ab}+P_{ab}\right)}$$

(4) $$StdErr=\sqrt{\frac{P_{aB}+R_{B\to b|a}\left(P_{AB}+P_{ab}\right)}{N\times P_{Ab}}}$$

Finally, Equation (5) was used to calculate the range of confidence values for the calculated attributable risks.

(5) $$\left[1-EXP\left(ln\left(1-R_{a}\right)-1.96\times StdErr\right)]\;to\;[1-EXP\left(ln\left(1-R_{a}\right)+1.96\times StdErr\right)\right]$$

The codependence information was integrated with the pDST metadata from the GMTV and annotation information from the Tuberculosis Drug Resistance Mutation Database (TBDream) database [38] as input for the creation of directed DR functional association networks using the program Cytoscape 3.7.2.

## 5. Conclusions

This study demonstrated that DR evolution is a complex process of adjusting the activities of many proteins including enzymes and transcription regulators through mutations at polymorphic loci of the bacterial genomes. Some of these mutations take place prior to the acquisition of DR mutations, creating in this way an epistatic background of DR development, whereas other mutations follow DR acquisition with an aim to reduce the fitness cost of DR mutations. It can be expected that the epistatic modifications do not cover all the mechanisms of the amelioration of genomes of multidrug-resistant pathogens. Epigenetic modifications consisting of an altered methylation of bacterial genomes may also play an important role and should be investigated in future studies. Therefore, uncovering the epistatic networks of DR by the methods of attributable risk statistics will shed light on the intrinsic mechanisms of stress adaptation exploited by bacteria and allow researchers to identify potentially dangerous pathogens prior to disease outbreaks by inspecting the profiles of genetic polymorphisms.

## Data Availability

Not applicable.
